# The Assessment of Sealants’ Effectiveness in Arresting Non-Cavitated Caries Lesion—A 24-Month Follow-Up

**DOI:** 10.3390/healthcare10091651

**Published:** 2022-08-30

**Authors:** Liana Beresescu, Mariana Păcurar, Cristina Ioana Bica, Alexandru Vlasa, Oana Elena Stoica, Timea Dako, Blanka Petcu, Daniela Esian

**Affiliations:** Faculty of Dental Medicine, University of Medicine and Pharmacy, Science, and Technology George Emil Palade, 540139 Târgu-Mureș, Romania

**Keywords:** resin-based sealant, retention, arresting caries lesions

## Abstract

Dental sealants are excellent means to prevent pits and fissure caries. Nowadays, the application of sealants is extended to therapeutic use in arresting non-cavitated carious lesions. This relatively new concept still lacks evidence to support its routine use. The aim of this study was to evaluate the effectiveness of a resin-based sealant applied on first permanent molars with carious lesions (ICDAS 1–3), in comparison with its effectiveness when applied on sound surfaces (ICDAS 0). Included in the study were 114 children aged between six and eight years old, with a high caries risk (according to the CAMBRA system), with all four permanent molars erupted and with deep pits and fissures. A total number of 407 molars were sealed and assessed. A total of 49 were excluded (they had caries, which according to the ICDAS II classification were classified with code 4–6 or had older sealants or fillings). Out of these 407 molars, 213 were sound (code 0) and 194 had caries lesions as follows: 56 teeth classified as code 1, 79 teeth classified as code 2, and 59 teeth classified as code 3 according to the ICDAS II classification. The retention of the sealant and carious lesions were assessed clinically at 6, 12, 18, and 24 months. Regarding sealant retention, a statistically significant difference (*p* < 0.05) among the two types of sealed teeth, sound (ICDAS 0) and decayed (ICDAS 3), showed at 18- and 24-month follow-up intervals. Regarding caries lesions, a statistically significant difference (*p* < 0.05) showed between sound (ICDAS 0) and decayed (ICDAS 3) molars at 24-month follow-up. Our study results supported the resin-based sealant effectiveness in arresting incipient carious lesions, which according to the ICDAS II classification have received codes 1 and 2 but did not support sealant effectiveness in arresting caries lesions classified according to the same classification with code 3.

## 1. Introduction

Dental caries is the most common dental disease affecting people of all ages. Even though its aetiology is well-known, it remains a significant worldwide disease and public health issue. The World Health Organization (WHO) estimated that it affects nearly 3.5 billion people [1]. In developing countries, the incidence and prevalence of dental caries reach very high levels due to the lack of preventive policies and programs, poor oral hygiene, and increased consumption of refined carbohydrates, especially sugar [1,2]. Even in developed countries where preventive programs have been running for many years, caries is the most common chronic disease of childhood [3]. Data from older and recent epidemiological studies show that 60–90% of carious lesions occur in the pits and fissures of permanent molars [4,5,6].

Due to the high prevalence and early onset of occlusal caries, its prevention has been one of the major goals in dentistry [7]. From all the methods and materials tested over time to protect these surfaces, the use of sealants has proven its effectiveness in preventing dental caries. After five decades of clinical use, pit and fissure sealants are now recognised as the most effective method for preventing occlusal caries in children [8,9,10,11]. Long-term studies have reported substantial reductions in the incidence of carious lesions and the risk of developing them. For example, one study by Wright et al. reported that children and adolescents who have sealants placed on sound pits and fissures of permanent molars have a 76% reduction in the risk of developing caries, compared with those that do not receive sealants. Moreover, after more than 7 years of follow-up studies, children who received sealants had a caries incidence of 29%, whereas those without sealants had a caries incidence of 74% [12]. Additionally, sealants have been shown to be much more effective in preventing pit and fissure caries compared to fluoride varnishes [13,14,15], the other professional method of caries prevention, which is highly recommended, but whose efficiency is clearly superior in preventing caries lesions on smooth surfaces.

Recently, advances in adhesive materials have facilitated the use of sealants to be extended to therapeutic uses such as managing caries lesions with no cavitation. This is a relatively new concept and still lacks evidence to support its routine use [7]. The hypothesis which underlies this concept is that the application of sealing material in incipient non-cavitated pit and fissure caries leads to the elimination of viable microorganisms and the arrest of caries. Recent studies have shown that lesions that are effectively sealed do not progress for many years [12,16,17,18,19]. The bacterial population recovered from pits and fissures was shown to decrease rapidly when it was sealed [20,21]. This decrease in microorganism number is probably due to the integrity of the seal between the material and the tooth surface, which does not permit the movement of fluids between the material and the hard dental structure [22,23]. Therefore, for the success of this method, the most important condition is a tight connection between sealing material and tooth surface [24]. Studies have shown that the sealing of non-cavitated lesions is not likely to result in progression if the sealant is intact [19,25]. Longitudinal data have shown that sealing with resin-based materials arrests non-cavitated carious lesions [18,19,25,26], but sealing with glass ionomer cement does not arrest the progression of these lesions [27,28]. This fact can be explained by superior retention of the composite resins compared to glass ionomer cement, retention which ensures the tight closure of the sealing material for a long period of time [29]. In addition, the acid-etching process used in the case of resin-based sealants can reduce the microorganism load, and the bacterial population that remains is not capable of maintaining the progression of caries [30,31].

The aim of this study was to evaluate the effectiveness of the therapeutic use of a resin-based sealant applied on recently erupted first permanent molars with carious lesions (ICDAS 1–3), in comparison with the effectiveness of the preventive application on sound surfaces (ICDAS 0), over a period of 24 months.

## 2. Materials and Methods

This study was conducted from July 2019 to July 2021 (24-month period), in a private medical centre, Denta Aur in Targu-Mures, Romania, with the clinical trial registration number 022/27.06.2019. The written informed consent of the parents of all children involved in the study was obtained before the beginning of the examinations.

All the examinations and procedures were performed by two experienced dentists, helped by a trained chair-side clinical assistant, following the 4-handed sitting dentistry model. Before the beginning of the examinations, the dentists completed an ICDAS II calibration course, assessing the condition of tooth surfaces and the presence of caries according to the International Caries Detection and Assessment System (ICDAS II) in a very similar way as previous researchers [32].

In order to recruit for our study, we examined 156 children (624 teeth) with ages between 6 and 8 years. The inclusion criteria were as follows: presence of four erupted permanent first molars without dental abnormalities, with deep pits and fissures, that were at caries risk. For assessing children caries risk, we used the Caries Management by Risk Assessment (CAMBRA) system [33]. A total of 42 children were excluded from the study because they did not meet the inclusion criteria, were not cooperative, or did not show up for all periodic check-ups. Therefore, 119 healthy cooperative children who were at high caries risk, with all four recently newly erupted permanent first molars (476 teeth), were included in the study.

All teeth were cleaned with a prophylaxis cup and a toothpaste without fluoride. The examination was made on clean and wet/dry teeth. We use a ball-ended explorer to remove any remaining plaque and debris and to check for surface contour, cavitation, fillings, or sealants.

Each occlusal surface was examined and received classification ranging from 0 up to 6 according to ICDAS II criteria [32] (Table 1):

Out of the total of 476 teeth, 427 molars were sealed by the operators; 49 teeth were excluded because they had caries, which according to the ICDAS II classification were classified with codes 4–6, or had older sealants or fillings. Of these 427 molars that we sealed, 225 were sound (code 0) and 202 had caries lesions as follows:

- A total of 64 teeth classified as code 1;
- A total of 79 teeth classified as code 2;
- A total of 59 teeth classified as code 3 according to the ICDAS II classification.

The sealing material used was a light-curing resin-based sealant, Helioseal F™, Ivoclar Vivadent Schaan, Liechtenstein. According to the manufacturer’s instructions, the application steps were: professional tooth cleaning, rinsing with water then air-drying, isolation with cotton rolls and air-drying, application of the enamel etching with phosphoric acid gel (37%) for 30 s, rinsing with water and air-drying, control of the acid-etched dental surface, bonding application, light curing of the bonding, sealant application, light curing of the sealant, control of marginal adaptation, and occlusion control.

The follow-up interval was every six months, over a period of 24 months. We evaluate sealant retention and the incidence of new caries lesions or the progression of the lesions that were already present. The integrity and marginal adaptation of the sealant were assessed through visual and tactile examination.

For assessing sealant retention, we used Simonsen’s criteria [34]:

- I: completely retained;
- II: partially retained;
- III: missing sealant.

### 2.1. Sample Size Determination

**Figure 1:**
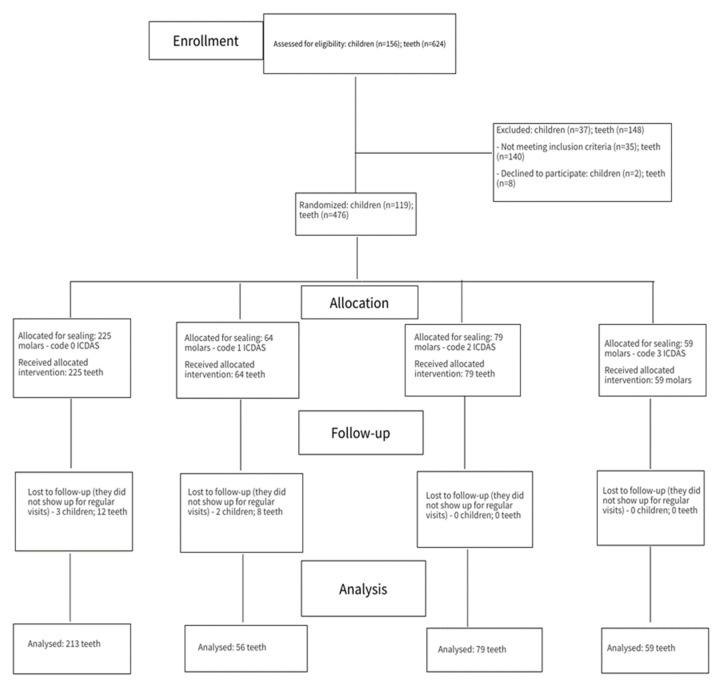
Consort flow diagram for the study.

The required sample size was determined to be 407 teeth using G-power software™, Heinrich Heine University, Dusseldorf, Germany, for Windows, for a power of 95% (α = 0.05, β = 0.05) (Figure 1).

### 2.2. Statistical Analysis

For evaluation of the categorical data, we used Fisher’s exact test and chi-squared test. The chosen significance level was set at 0.05, and *p* was considered significant when *p* ≤ 0.05. All data were recorded using GraphPad Prism™ V6.01 software for Windows™ 2017. 

## 3. Results

**Figure 2:**
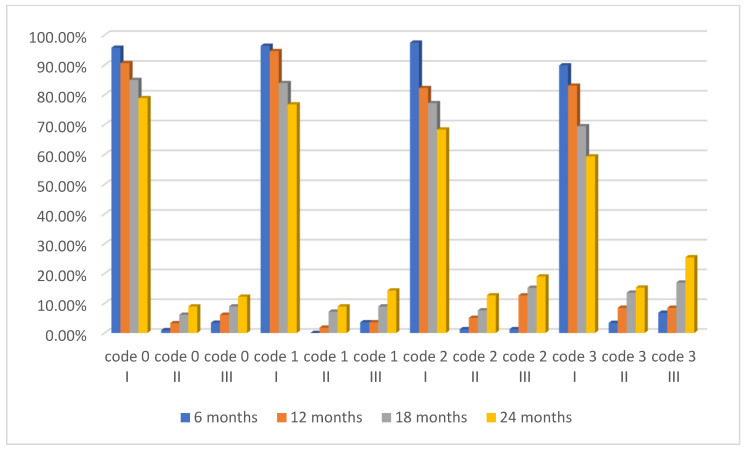
Retention based on Simonsen’s criteria at each follow-up interval.

For 5 children, we could not perform all the periodic check-ups, so we had to exclude them throughout the study period. The final sample of the study included 114 children and 456 first permanent molars which were sealed and assessed. Of these molars, 213 were sound (ICDAS 0) and 194 had caries lesions (ICDAS 1–3). The follow-up intervals were at 6, 12, 18, and 24 months. All the obtained data were systematised in tables (Table 2, Table 3, Table 4, Table 5, Table 6, Table 7, Table 8 and Table 9) and one figure (Figure 2).

The 6- and 12-month follow-up intervals showed no statistically significant differences (*p* > 0.05) regarding sealant retention between the two types of sealed teeth, sound (ICDAS 0) and decayed (ICDAS 1–3) (Table 2 and Table 3). The 18-month follow-up showed statistically significant differences (*p* = 0.02) between sound (ICDAS 0) and decayed (ICDAS 3) sealed teeth (Table 4). The 24-month follow-up showed statistically significant differences (*p* = 0.0086) regarding sealant retention, between sound (ICDAS 0) and decayed (ICDAS 3) molars (Table 5).

The 6, 12, and 18-month follow-up assessments showed no statistically significant differences (*p* > 0.5) between the two types of sealed teeth concerning carious lesion development (Table 6, Table 7 and Table 8).

The 24-month follow-up assessment showed statistically significant differences (*p* = 0.0478) concerning carious lesion development only between two types of sealed teeth—sound (ICDAS 0) and with caries lesion (ICDAS 3) (Table 9).

Some of the carious lesions initially classified with codes 0, 1, or 2 according to ICDAS II classification required the reapplication of a new sealant on the 6, 12, 18, and 24-month follow-up assessments. Three of these lesions progressed to code 3. None of these lesions progressed to code 4–6, which would indicate the application of fillings.

Some of the carious lesions initially classified with code 3 according to ICDAS II criteria required the reapplication of sealants at 6- and 12-month assessments. At 18- and 24-month follow-up assessments, 4 and 9 lesions, respectively, required the application of fillings, due to their transformation into lesions classified with codes 4 or 5.

## 4. Discussion

The idea of sealing and arresting a carious lesion in the pits and fissures is not new, as many researchers have pointed this out since the 1970s. Handelman was the first to support the possibility of arresting dental caries with the help of sealants and without the need for an invasive intervention to remove the caries lesion [34]. Since then, several researchers have studied this concept, and the results have suggested that the carious lesion can be arrested by using proper materials and techniques [21,25,35,36,37].

Analysing these studies’ results, we found out that the information provided is very general, and it is difficult to draw exact conclusions based on which practitioners have clear recommendations about which caries lesions could be sealed and arrested [38].

Out of the desire to clarify certain aspects regarding the situations in which sealants have the highest efficiency, related to the depth of carious lesions that can be arrested with dental materials, in our study, we used precise criteria. The children participating in the research were divided according to the CAMBRA classification [34] in the category of high caries risk children.

It is very important to notice that not every tooth would necessarily become affected, so the selection of children and teeth which must receive a sealant is one of the most important steps. For the sealants to be effective, they have to be placed in only those pits and fissure that are at caries risk. Regarding caries risk, one of the best predictors of caries in permanent dentition is the presence of carious lesions in temporary dentition. This is due to the accumulation of factors such as the high level of cariogenic bacteria found in the mouth of these children, poor oral hygiene, and carbohydrate consumption [39,40]. Moreover, another factor, which is also a good predictor of dental caries, is the depth of pits and fissures [41,42,43,44,45].

Sealants placed only on children (teeth) that are at caries risk have shown a positive outcome (cost-effective and less time-consuming) in clinical practice, especially in public health programs [46,47].

The results of our study are similar to those of studies from the literature, supporting the effectiveness of sealants in the prevention of caries in the pits and fissures of sound teeth, classified according to the International Caries Detection and Assessment System (ICDAS II) with code 0 [8,9,10,11,12], and also supporting their effectiveness in arresting incipient carious lesions, which according to the same classification have received codes 1 and 2 [12,16,17,18,19,25].

Regarding the lesions classified according to ICDAS II with code 3, our study results showed that sealants are not so effective in arresting this stage of carious lesion. This may be due to the lower retention of the sealant when applied on these lesions, which leads to a higher rate of reseals and the progression of carious lesions that have lost the covering material. It is generally accepted that the efficacy of resin-based sealants in preventing caries is based on good retention [48,49,50].

The poor retention of sealants in the case of demineralised enamel and dentin which surround caries fissures has been also reported by other researchers. These demineralised tissues have reduced adhesive properties [51,52] and could contain impossible-to-remove dental plaque, thus preventing a sealant from adapting properly [53]. Even though contemporary protocols for the application of sealants support the use of an adhesive to enhance retention, studies have shown that in the case of caries fissures, its use does not influence the microleakage and the penetration ability [54,55], with carious pits and fissures showing significantly more microleakage and insufficient sealant penetration depth than sound fissures [56]. Therefore, this compromised bonding allows saliva and microorganisms to infiltrate the spaces between the dental structure and sealant to undermine the material, cause its failure, and cause caries progression [57].

### Limitations of the Study

Because of the small sample size and short period of time, the results of our study must be interpreted with caution.

## 5. Conclusions

The results of this study supported the effectiveness of dental sealant materials in the prevention of caries in the pits and fissures of sound teeth, classified according to ICDAS II with code 0, and also supported their effectiveness in arresting incipient carious lesions classified according to the same classification with codes 1 and 2.

Our study results did not support sealant effectiveness in arresting caries lesions classified according to ICDAS II with code 3.

## Figures and Tables

**Table 1:** International Caries Detection and Assessment System.

Code 0	sound tooth surface; no evidence of caries after prolonged air drying (5 s)
Code 1	first visual change in enamel: opacity or discolouration (white or brown) is visible at the entrance to the pit or fissure after prolonged air drying, which is not or hardly seen on wet surface
Code 2	distinct visual change in enamel: opacity or discolouration distinctly visible at the entrance to the pit and fissure when wet, lesion must still be visible when dry
Code 3	localised enamel breakdown due to caries with no visible dentine or underlying shadow: opacity or discolouration wider than the natural fissure/fossa when wet and after prolonged air drying
Code 4	underlying dark shadow from dentine with or without localised enamel breakdown
Code 5	distinct cavity with visible dentine: visual evidence of demineralisation and dentine exposed
Code 6	extensive distinct cavity with visible dentine and more than half of the surface involved

**Table 2:** Retention based on Simonsen’s criteria at the 6-month follow-up.

6 Months	I	II	III	Total	*p* Value
code 0 ICDAS	95.77% (204)	0.93% (2)	3.43% (7)	213	
code 1 ICDAS	96.42% (54)	0	3.57% (2)	56	*p* = 0.7639
code 2 ICDAS	97.46% (77)	1.26% (1)	1.26% (1)	79	*p* = 0.6263
code 3 ICDAS	89.83% (53)	3.38% (2)	6.77% (4)	59	*p* = 0.1778

**Table 3:** Retention based on Simonsen’s criteria at the 12-month follow-up.

12 Months	I	II	III	Total	*p* Value
code 0 ICDAS	90.61% (193)	3.28% (7)	6.10% (13)	213	
code 1 ICDAS	94.64% (53)	1.78% (1)	3.57% (2)	56	*p* = 0.6299
code 2 ICDAS	82.27% (65)	5.06% (4)	12.6% (10)	79	*p* = 0.1301
code 3 ICDAS	83.05% (49)	8.47% (5)	8.47% (5)	59	*p* = 0.1731

**Table 4:** Retention based on Simonsen’s criteria at the 18-month follow-up.

18 Months	I	II	III	Total	*p* Value
code 0 ICDAS	84.97% (181)	6.10% (13)	8.92% (19)	213	
code 1 ICDAS	83.92% (47)	7.14% (4)	8.92% (5)	56	*p* = 0.9600
code 2 ICDAS	77.21% (61)	7.59% (6)	15.18% (12)	79	*p* = 0.2529
code 3 ICDAS	69.47% (41)	13.55% (8)	16.94% (10)	59	*p* = 0.0238

**Table 5:** Retention based on Simonsen’s criteria at the 24-month follow-up.

24 Months	I	II	III	Total	*p* Value
code 0 ICDAS	78.87% (168)	8.92% (19)	12.20% (26)	213	
code 1 ICDAS	76.78% (43)	8.92% (5)	14.28% (8)	56	*p* = 0.9156
code 2 ICDAS	68.35% (54)	12.65% (10)	18.98% (15)	79	*p* = 0.1706
code 3 ICDAS	59.32% (35)	15.25% (9)	25.42% (15)	59	*p* = 0.0086

**Table 6:** Presence of carious lesions at 6 months.

6 Months	Yes	No	Total	*p* Value
code 0 ICDAS	0	213	213	-
code 1 ICDAS	0	56	56	-
code 2 ICDAS	0	79	79	-
code 3 ICDAS	1.69% (1)	98.30% (58)	59	-

**Table 7:** Presence of carious lesions at 12 months.

6 Months	Yes	No	Total	*p* Value
code 0 ICDAS	4.22% (9)	95.77% (204)	213	-
code 1 ICDAS	3.57% (2)	96.42% (56)	56	*p* = 1.0000
code 2 ICDAS	3.79% (3)	96.20% (76)	79	*p* = 1.0000
code 3 ICDAS	5.08% (3)	94.91% (56)	59	*p* = 0.7268

**Table 8:** Presence of carious lesions at 18 months.

18 Months	Yes	No	Total	*p* Value
code 0 ICDAS	6.57% (14)	93.42% (199)	213	
code 1 ICDAS	5.35% (3)	94.64% (53)	56	*p* = 1.0000
code 2 ICDAS	6.32% (5)	93.67% (74)	79	*p* = 1.0000
code 3 ICDAS	10.16% (6)	89.83% (53)	59	*p* = 0.5126

**Table 9:** Presence of carious lesions at 24 months.

24 Months	Yes	No	Total	*p* Value
code 0 ICDAS	9.38% (20)	90.61% (193)	213	
code 1 ICDAS	10.71% (6)	89.28% (50)	56	*p* = 0.9646
code 2 ICDAS	10.12% (8)	89.87% (71)	79	*p* = 0.8493
code 3 ICDAS	18. 64% (11)	81.35% (48)	59	*p* = 0.0478

## Data Availability

Not applicable.
